# The impact of sequence database choice on metaproteomic results in gut microbiota studies

**DOI:** 10.1186/s40168-016-0196-8

**Published:** 2016-09-27

**Authors:** Alessandro Tanca, Antonio Palomba, Cristina Fraumene, Daniela Pagnozzi, Valeria Manghina, Massimo Deligios, Thilo Muth, Erdmann Rapp, Lennart Martens, Maria Filippa Addis, Sergio Uzzau

**Affiliations:** 1 Porto Conte Ricerche, Science and Technology Park of Sardinia, Tramariglio, Alghero, Italy; 2 Department of Biomedical Sciences, University of Sassari, Sassari, Italy; 3 Max Planck Institute for Dynamics of Complex Technical Systems, Magdeburg, Germany; 4 Research Group Bioinformatics (NG 4), Robert Koch Institute, Berlin, Germany; 5 Department of Biochemistry, Ghent University, Ghent, Belgium; 6 Medical Biotechnology Center, VIB, Ghent, Belgium; 7 Bioinformatics Institute Ghent, Ghent University, Zwijnaarde, Ghent, Belgium

**Keywords:** Bioinformatics, Gut microbiota, Mass spectrometry, Metagenomics, Metaproteomics

## Abstract

**Background:**

Elucidating the role of gut microbiota in physiological and pathological processes has recently emerged as a key research aim in life sciences. In this respect, metaproteomics, the study of the whole protein complement of a microbial community, can provide a unique contribution by revealing which functions are actually being expressed by specific microbial taxa. However, its wide application to gut microbiota research has been hindered by challenges in data analysis, especially related to the choice of the proper sequence databases for protein identification.

**Results:**

Here, we present a systematic investigation of variables concerning database construction and annotation and evaluate their impact on human and mouse gut metaproteomic results. We found that both publicly available and experimental metagenomic databases lead to the identification of unique peptide assortments, suggesting parallel database searches as a mean to gain more complete information. In particular, the contribution of experimental metagenomic databases was revealed to be mandatory when dealing with mouse samples. Moreover, the use of a “merged” database, containing all metagenomic sequences from the population under study, was found to be generally preferable over the use of sample-matched databases. We also observed that taxonomic and functional results are strongly database-dependent, in particular when analyzing the mouse gut microbiota. As a striking example, the *Firmicutes*/*Bacteroidetes* ratio varied up to tenfold depending on the database used. Finally, assembling reads into longer contigs provided significant advantages in terms of functional annotation yields.

**Conclusions:**

This study contributes to identify host- and database-specific biases which need to be taken into account in a metaproteomic experiment, providing meaningful insights on how to design gut microbiota studies and to perform metaproteomic data analysis. In particular, the use of multiple databases and annotation tools has to be encouraged, even though this requires appropriate bioinformatic resources.

**Electronic supplementary material:**

The online version of this article (doi:10.1186/s40168-016-0196-8) contains supplementary material, which is available to authorized users.

## Background

The interest in studying the gut microbiota has seen a tremendous rise over the past years, due to the increasing recognition of its involvement in a wealth of physiological functions and multifactorial diseases [1]. Consequently, gut microbiota research is shifting from a mere description of the taxonomic distribution to a more comprehensive exploration of a functional potential and activity of the microbial community [2, 3]. To this purpose, metaproteomics, i.e., the study of the whole protein complement of a microbial community, can reveal which functions are actually being expressed by the gut microbiota in response to host, diet, or other environmental stimuli [4–6].

In spite of the recent development of dedicated tools enabling the reliable and integrated detection of taxonomic and functional features of a metaproteome, metaproteomic data analysis remains a challenge [7–9]. One of its most critical steps is the choice of proper protein databases (DBs) for the identification of mass spectra. Protein identification, in fact, essentially relies on matching experimental mass spectra, generated from the sample under study, with theoretical spectra, typically generated in silico from a sequence DB. Since a gut microbiota may contain up to over a thousand different microbial species, and its composition can vary considerably among individuals, the selection of a well-suited DB is rather challenging. In addition, the use of large sequence search spaces, without any prior restriction to selected sequences/taxa, poses several FDR-related issues which impair the identification rate [10, 11]. Even more importantly, despite the impressive efforts undertaken in human metagenome research, for most microbial species inhabiting the gut of higher animals few or no protein sequences are present in public repositories [1]. Cross-species identification is often possible, owing to the high sequence similarity among orthologous genes from closely related microorganisms [12], but a single amino acid change is sufficient to hamper peptide-to-spectrum matching, making protein identification impossible. Iterative and error-tolerant DB searches, as well as DB-independent de novo (peptide) sequencing, have been proposed as improvements or alternatives to classical DB search [11, 13–16]. However, an optimized and standardized mass spectrometry (MS) data analysis pipeline for metaproteomics is not yet available.

An alternative way to enhance sensitivity of metaproteomic analysis builds on its specific integration with metagenomics, which can be established at different levels: first, when the taxonomic structure of the microbiota under study is known (typically based on 16S rDNA gene sequencing data), a “pseudo-metagenome” can be assembled, that is, a DB containing all publicly available sequences for the taxa that are predicted to form that particular microbiota [17, 18]; second, if metagenome sequences can be obtained for all, or a subset of, the samples under study, these can be translated, annotated, and used as DB. A few recent studies tried to explore and benchmark these integration strategies, focusing on metagenomes from human fecal samples obtained using the 454 sequencing technology [19], or on mock microbial mixtures analyzed by means of the Illumina sequencing technology [15]. However, a systematic investigation aimed at elucidating the influence of processing, combination and, most importantly, taxonomic and functional annotation of metagenomics-based DB sequences on gut metaproteomic results is still lacking.

In keeping with this, we describe a systematic evaluation of the impact of sequence DBs on gut metaproteomic results, providing useful insights on how to design gut microbiota studies. In particular, we aim to investigate: (i) what is the best strategy between using experimental metagenomic and publicly available DBs, and to what extent their outcomes are different; (ii) when constructing a metagenomic DB, if sequence assembly into contigs provides additional information in comparison with unassembled reads; (iii) if different DB types provide different yields/outcomes in terms of taxonomic and functional annotation; and (iv) if the answers to the previous questions change when analyzing stool samples from mouse models instead of from human patients/volunteers. By tackling these issues, we seek to identify biases and critical points in metaproteomic data analysis, which need to be taken into consideration when planning metaproteomic experiments in the context of large cross-sectional studies involving human subjects or animal models.

## Results

### Experimental metagenomic databases and UniProt-based databases lead to identification of different gut microbiota peptide assortments

**Fig. 1:**
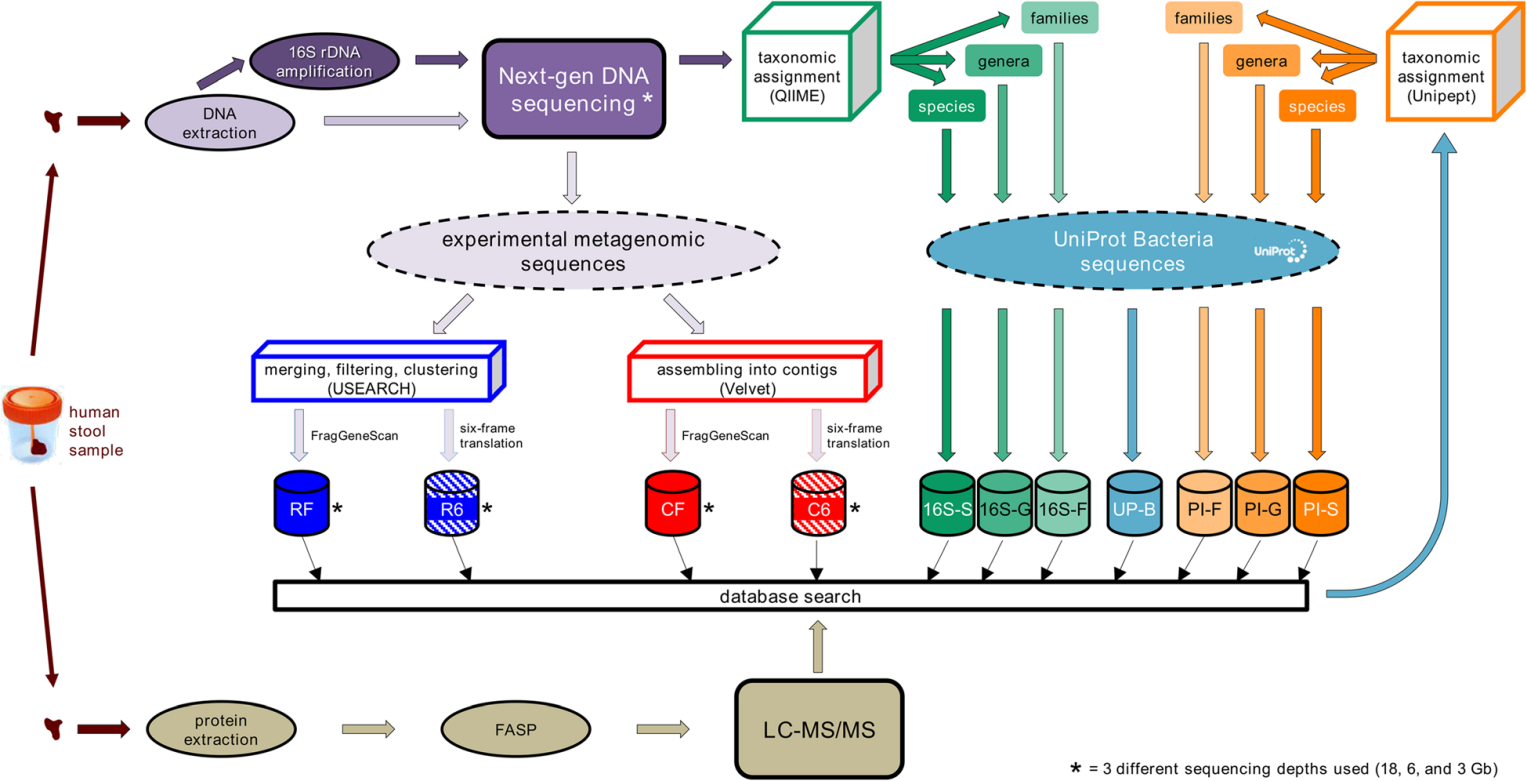
Design of the human stool sample experiment. Database-related acronyms contained into colored cylinders correspond to those indicated in Fig. 2

The main aim of this study was to perform a systematic comparison of the main DB types which can be used in a metaproteomic study, according to both sequencing-based and sequencing-independent approaches. To this purpose, we chose to conduct a preliminary investigation of the impact of several DB construction variables using a single human stool sample. Hence, as illustrated in Fig. 1, the fecal material collected from a healthy human volunteer was split into two equal portions, to be processed in parallel according to a metagenomic and a metaproteomic approach, and therefore to generate a wide gamut of sequence DBs. In total, we generated 19 different DBs, whose characteristics are presented in Table 1. Among them, 12 were experimental metagenomic DBs (i.e., obtained by metagenomic sequencing of the same individual and analyzed by metaproteomics; MG-DBs), differing based on common sequencing and sequence processing variables, namely sequencing depth, read assembly level, and ORF finding method; three were “pseudo-metagenomes”, containing UniProt sequences taxonomically filtered based on 16S-rDNA sequencing data at thee different taxonomic levels (species, genus, and family); four were sequencing-independent DBs, with the first comprising the entire set of bacterial sequences deposited in UniProt (UP-B) at the time of the analysis, and the remaining three constructed according to a previously described taxonomy-based proteomic iterative (PI) strategy [15, 20] (see Methods for further details). MS data obtained from the human stool sample were then searched against all sequence DBs in parallel using three different bioinformatics platforms and two FDR thresholds (namely, 1 and 5 %), in order to rule out potential search engine- or statistics-based biases.

**Table 1:** Characteristics of sequence databases used in this study

Source	Depth	Sample	Type	Further information	Sequences
UniProt	Bacteria				72,669,092
UniProt	Bacteria	Human_0	16S	Family	3,590,268
UniProt	Bacteria	Human_0	16S	Genus	2,422,588
UniProt	Bacteria	Human_0	16S	Species	610,219
UniProt	Bacteria	Human_0	PI	Family—MPA	3,095,210
UniProt	Bacteria	Human_0	PI	Genus—MPA	2,317,980
UniProt	Bacteria	Human_0	PI	Species—MPA	178,908
UniProt	Bacteria	Human_0	PI	Family—MQ	2,717,146
UniProt	Bacteria	Human_0	PI	Genus—MQ	2,162,216
UniProt	Bacteria	Human_0	PI	Species—MQ	190,367
UniProt	Bacteria	Human_0	PI	Family—PD	3,173,395
UniProt	Bacteria	Human_0	PI	Genus—PD	2,602,972
UniProt	Bacteria	Human_0	PI	Species—PD	225,013
Metagenome	18 Mbps	Human_0	Reads	FragGeneScan	5,130,156
Metagenome	18 Mbps	Human_0	Reads	Six-frame translation	27,651,587
Metagenome	18 Mbps	Human_0	Contigs	FragGeneScan	224,163
Metagenome	18 Mbps	Human_0	Contigs	Six-frame translation	4,353,453
Metagenome	6 Mbps	Human_0	Reads	FragGeneScan	3,423,708
Metagenome	6 Mbps	Human_0	Reads	Six-frame translation	18,350,764
Metagenome	6 Mbps	Human_0	Contigs	FragGeneScan	192,582
Metagenome	6 Mbps	Human_0	Contigs	Six-frame translation	3,205,893
Metagenome	3 Mbps	Human_0	Reads	FragGeneScan	3,294,112
Metagenome	3 Mbps	Human_0	Reads	Six-frame translation	17,340,365
Metagenome	3 Mbps	Human_0	Contigs	FragGeneScan	109,233
Metagenome	3 Mbps	Human_0	Contigs	Six-frame translation	2,447,096
Metagenome	6 Mbps	Human_1	Contigs	FragGeneScan	101,903
Metagenome	6 Mbps	Human_1	Reads	FragGeneScan	1,288,040
Metagenome	6 Mbps	Human_2	Contigs	FragGeneScan	97,532
Metagenome	6 Mbps	Human_2	Reads	FragGeneScan	953,147
Metagenome	6 Mbps	Human_3	Contigs	FragGeneScan	60,517
Metagenome	6 Mbps	Human_3	Reads	FragGeneScan	625,354
Metagenome	6 Mbps	Mouse_1	Contigs	FragGeneScan	14,743
Metagenome	6 Mbps	Mouse_1	Reads	FragGeneScan	510,364
Metagenome	6 Mbps	Mouse_2	Contigs	FragGeneScan	88,366
Metagenome	6 Mbps	Mouse_2	Reads	FragGeneScan	600,697
Metagenome	6 Mbps	Mouse_3	Contigs	FragGeneScan	18,112
Metagenome	6 Mbps	Mouse_3	Reads	FragGeneScan	329,261

**Fig. 2:**
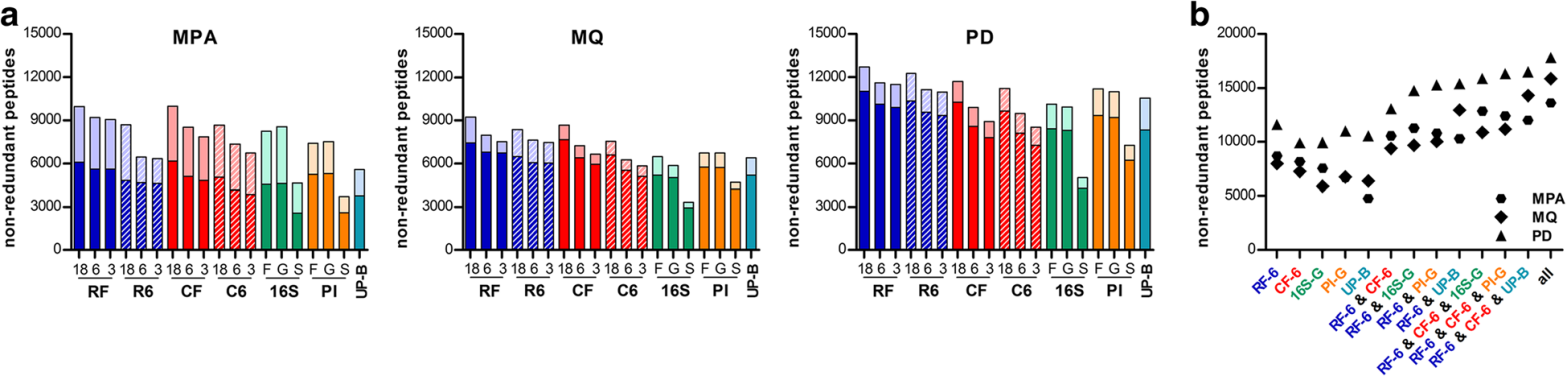
Peptide identification metrics in human gut metaproteomic data obtained using different databases. **a** Non-redundant peptides identified by searching the MS spectra obtained from a human stool sample against 19 different types of sequence DBs. Each graph illustrates the results achieved using a different bioinformatic platform, namely (from *left to right*) MetaProteomeAnalyzer (MPA), MaxQuant (MQ), and Proteome Discoverer (PD). RF and R6 (in *blue*) represent sequence DBs based on metagenomic reads processed by FragGeneScan or six-frame translation, respectively, while CF and C6 (in *red*) represent sequence DBs based on metagenomic assembled contigs processed by FragGeneScan or six-frame translation, respectively; 18, 6, and 3 are referred to the sequencing depth (in gigabases). Data from UniProt-based DBs are depicted in *green* (“pseudo-metagenomes” based on taxa found by 16S rDNA gene analysis), *orange* (sequences selected based on taxa found by a proteomic iterative approach, PI; see Methods for further details), and *turquoise* (all bacterial sequences); *F*, *G* and *S* are referred to the level of taxonomic filtering (family, genus, and species, respectively). Each column in the histograms contains a darker (identifications with FDR <1 %) and a lighter part (additional identifications with FDR <5 %). **b** Total peptide identifications obtained using five representative DBs and related multiple DB searches (*ampersand* indicates merging results from different DBs). All searches were run in triplicate using the three abovementioned bioinformatic platforms

Peptide identification metrics obtained with all sequence DBs are comparatively shown in Fig. 2. Concerning open reading frame (ORF) finding methods and read assembly level, six-frame translation did not provide significant advantages over FragGeneScan, while read-based MG-DBs slightly outperformed contig-based ones, although the latter provided a specific contribution in unique peptides (Additional file 1: Figure S1). Taxonomic filters at family and genus levels performed clearly better than those at species level for all UniProt-based DBs (Additional file 2: Figure S2). Overall, regardless of the bioinformatics platform used, MG-DBs performed better than UniProt-based DBs. Moreover, sequencing depth was observed to have a linear relationship with MG-DBs metrics, with a stronger impact on contig-based DBs. However, each DB family provided a significant percentage of unique peptides (up to over 50 %; Fig. 2b and Additional file 3: Figure S3).

### UniProt-based databases show severe limitations for gut metaproteomics of mouse models, but not of human subjects

A number of experimental mice models have been exploited to date, enabling the simulation of extreme changes in the microbiota. Environmental and genetic effects are responsible for taxonomic differences between the gut microbiome of mice raised in different laboratory and/or obtained by different vendors [21, 22]. Hence, the gut microbiota of a given experimental group of mice can include subsets of bacterial species and strains with no or poor sequencing background in public DBs, particularly at low taxonomic levels. We therefore extended our metaproteogenomic analysis to mouse stool samples. Based on the aforementioned preliminary results, we decided to focus on three main DB types (namely, reads- and contig-based MG-DBs and UP-B), keeping sequencing depth (6 Gb), ORF finding method (FragGeneScan), search engine (Sequest-HT), and FDR threshold (1 % according to Percolator) unvaried. For both humans and mice, we analyzed a small population (three samples per host species), in order to take individual variability into account. Each stool sample was split into two portions of equal size and subjected in parallel to metagenomic and metaproteomic analyses, as described above. MG-DBs were constructed by merging all sequences from each population (DB characteristics are provided in Table 1).

**Fig. 3:**
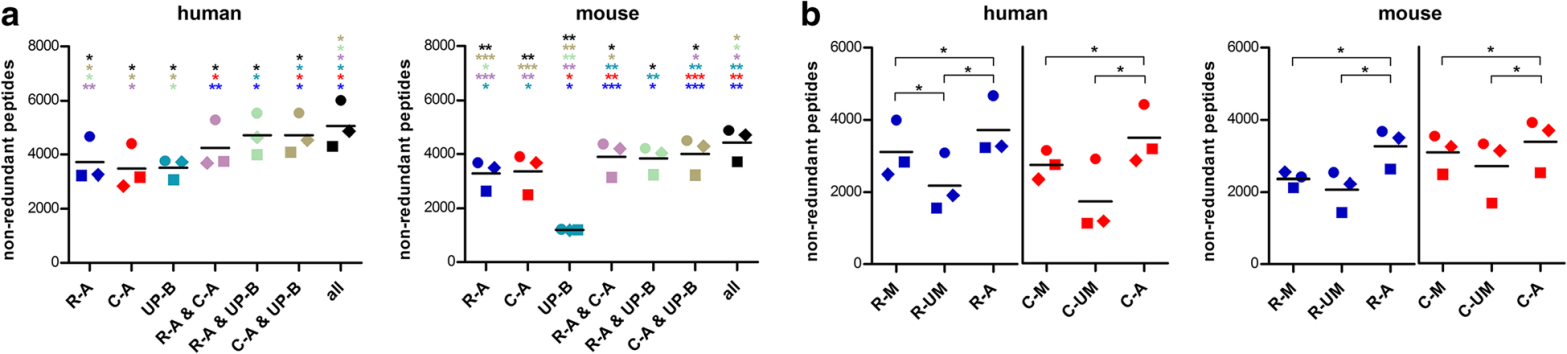
Peptide identification metrics in human and mouse gut metaproteomic data obtained using different databases. **a** Results obtained with human (*N* = 3, *left*) and mouse (*N* = 3, *right*) samples, with each *dot* representing a single sample. *R-A* and *C-A* are referred to DBs constructed with metagenomic reads and contigs from all samples, respectively. UP-B indicates a UniProt-based DB containing all bacterial entries. *Ampersand* indicates merging results from parallel DB searches. *Asterisks* indicate significantly different peptide identification yields between DBs (*p < 0.05; **p < 0.01; ***p < 0.001; paired *t* test), with the asterisk color corresponding to the DB to which the comparison is referred (e.g., the *green asterisk over blue dots* indicates significance of the difference between “R-A” and “R-A & UP-B”). **b** Comparison of metagenomic DB construction strategies applied to human (*N* = 3, *left*) and mouse (*N* = 3, *right*) samples, with each dot representing a single sample. “R” and “C” refer to reads (*blue*) and contigs (*red*), respectively. Matched DBs (sequences from the gut metagenome of the same subject analyzed by metaproteomics) are indicated with “*-M*,” unmatched DBs (sequences from a gut metagenome of another subject of the same host species) are indicated with “*-UM*;” merged DBs (sequences from gut metagenomes of all subjects analyzed for that host species) are indicated with *“-A.*” *p < 0.05 (paired *t* test)

As a striking result, for all mouse samples, UP-B performed dramatically worse than MG-DBs, while the number of identified peptides between the two DB families were similar in human samples (Fig. 3a). Again, each DB type provided a significant amount of unique peptides in both species, confirming multiple DB searches as a mean to considerably increase the number of peptide identifications.

### Databases merging sequences from different individuals lead to higher peptide identification yields when compared to sample-matched databases

The time-consuming and costly generation of MG-DBs requires the careful evaluation of options aimed at reducing the number of metagenomic sequencing runs in any metaproteomic study. Therefore, given the availability of metagenomic sequences from each of the human and mice samples, we addressed the question on whether using a sample-matched DB (i.e., the gut metagenome of the very same subject analyzed by metaproteomics)—as suggested by previous studies [19]—could represent a better strategy than using a larger DB containing all metagenomic sequences from the population under study. Further, we sought to determine if using a sample-matched DB could provide significant advantages on using another gut metagenome from the same population (i.e., an “unmatched” DB). As shown in Fig. 3b, both sample-matched and unmatched DBs provided a significantly lower amount of peptide identifications when compared to the DBs containing all metagenomic sequences from the population (both for human and mouse); moreover, the sample-matched DB significantly outperformed the corresponding unmatched DB only when comparing human read-based MG-DBs (for the other DB types, the slight differences measured were not significant).

According to these findings, reads- and contig-based MG-DBs containing all metagenomic sequences from the population and UP-B were selected for all subsequent analyses.

### Taxonomic annotation in mouse gut metaproteomics shows strong database- and classification algorithm-dependent biases

**Fig. 4:**
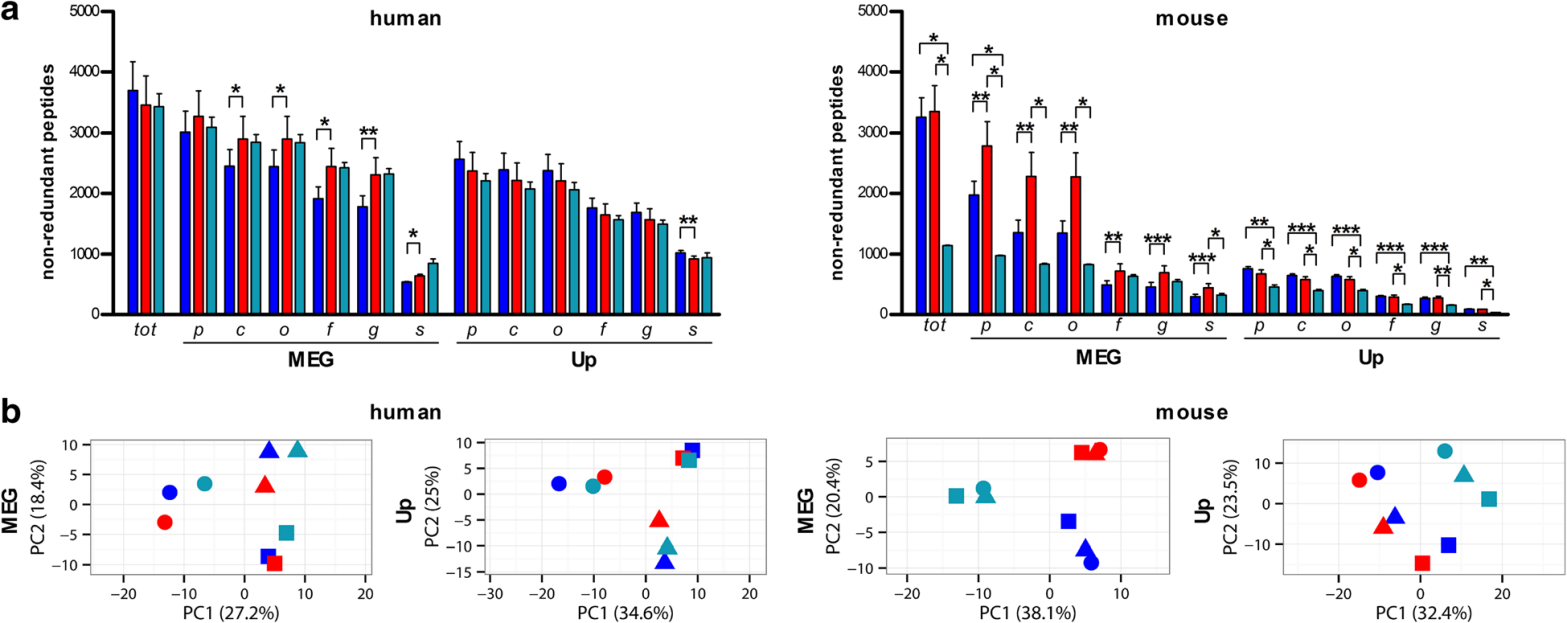
Taxonomic annotation of human and mouse gut metaproteomic data obtained using different databases. DBs were made up of reads (*dark blue*, R-A) or contigs (*red*, C-A) from gut metagenomes of all subjects analyzed for each host species, or all bacterial sequences deposited in UniProt (*turquoise*, UP-B). The annotation tools used were MEGAN (MEG) and Unipept (Up). **a** Histograms showing the mean number (*N* = 3, with *error bar* indicating standard error of the mean) of non-redundant peptides identified (*tot*) and annotated at different taxonomic levels (*p* phylum; *c* class; *o* order; *f* family; *g* genus; *s* species) in human (*left*) and mouse (*right*) samples. **b** PCA plots of taxa abundances, with each *dot* indicating a different human (*left*) or mouse (*right*) subject

As a further step, we performed taxonomic annotation by means of two established tools which employ a lowest common ancestor (LCA) algorithm: first, MEGAN was used to carry out LCA classification of read, contig, and protein sequences (identified from each respective DB) based on results of sequence alignment versus NCBI-nr entries; second, Unipept was employed to perform LCA classification of tryptic peptide sequences (obtained from each DB search) based on full homology with UniProt entries. As shown in Fig. 4a, UP-B generally provided a poorer annotation performance compared to MG-DBs, with small differences in humans and greater disparity in mice (especially down to the order level). Moreover, when focusing on MG-DBs, a global decrease in peptide annotation yield was found in mouse when compared to human samples. Concerning the annotation tools, MEGAN reached higher taxonomic annotation yields compared to Unipept, for both host species and all DB types. A strong impact of sequence length could be also observed for MEGAN, with the contig-based DBs (average sequence length of 209 and 344 amino acids in humans and mice, respectively) significantly outperforming the read-based DBs (average sequence length of 48 and 45 amino acids in humans and mice, respectively). Alpha-diversity calculation also revealed DB- and annotation tool-dependent biases (Additional file 4: Figure S4). MEGAN classification considerably enhanced the divergence between human (higher) and mouse (lower) gut metaproteome alpha-diversity, and generally higher alpha-diversity values were retrieved from Unipept data compared to MEGAN results. Even more intriguingly, according to the principal component analysis (PCA) of taxa abundances, human and mouse data clustered in a completely different fashion, namely according to individuals and to DBs, respectively (Fig. 4b). This clearly indicates a strong DB-dependent bias in taxonomic annotation of mouse gut metaproteomic data.

### Metaproteomic *Firmicutes*/*Bacteroidetes* ratios are considerably influenced by the sequence database type used

**Fig. 5:**
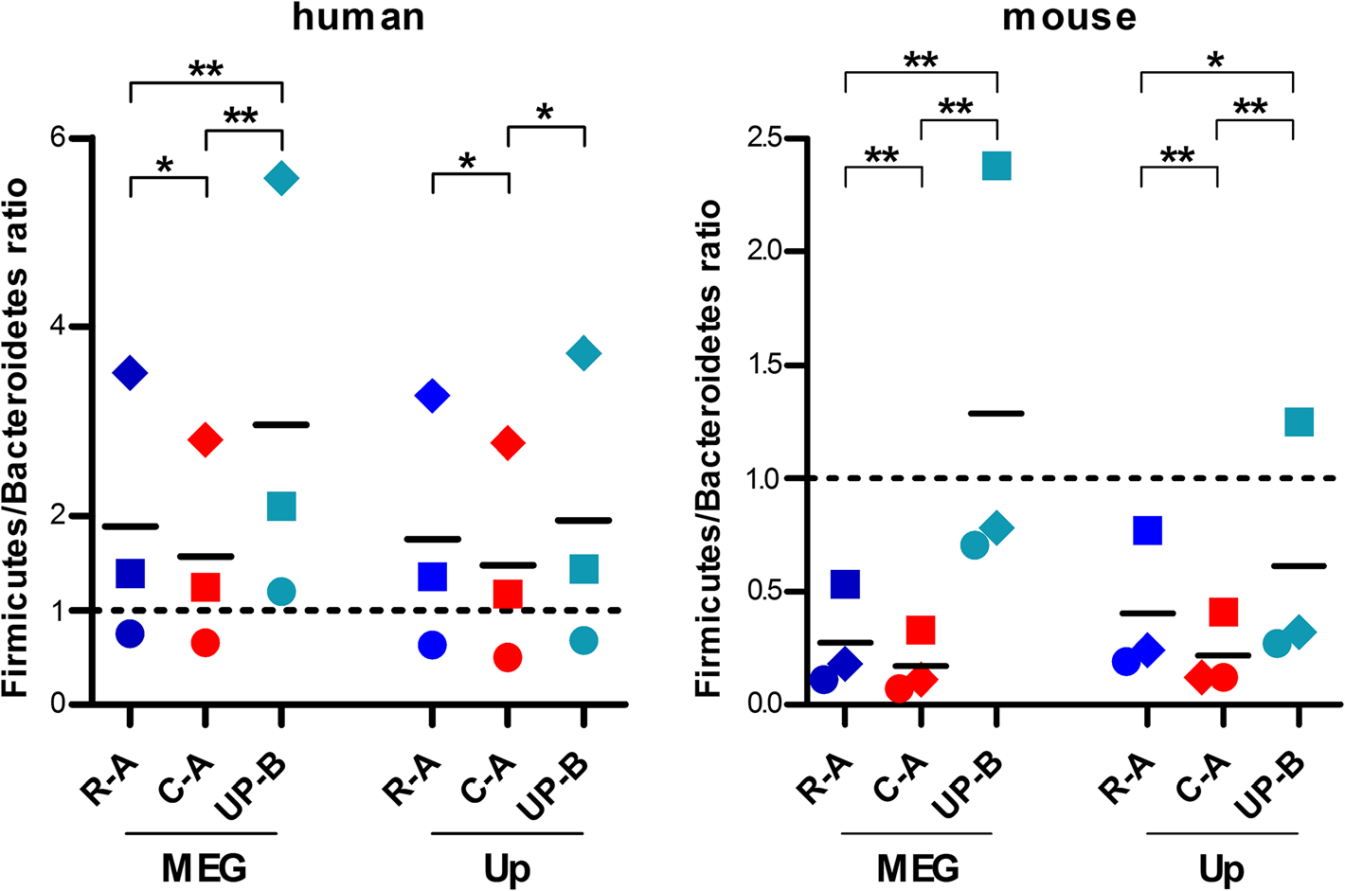
*Firmicutes*/*Bacteroidetes* ratio in human and mouse subjects measured using different databases. Results obtained with human (*N* = 3, *left*) and mouse (*N* = 3, *right*) samples, with each *dot* representing a single sample. DBs were made up of reads (*dark blue*, R-A) or contigs (*red*, C-A) from gut metagenomes of all subjects analyzed for each host species, or all bacterial sequences deposited in UniProt (*turquoise*, UP-B). The annotation tools used were MEGAN (MEG) and Unipept (Up). *p < 0.05; **p < 0.01; ***p < 0.001 (paired *t* test)

Taxa abundances were then subjected to LEfSe differential analysis, in order to find taxa specifically enriched/depleted when using a particular DB type. To gain insight into the differential taxonomic information achievable with each DB, we distinguished between “qualitatively differential taxa” (i.e., always present when using a given DB and completely absent when using another DB) and “quantitatively differential taxa” (i.e., all the remaining differential taxa). As a result (Additional file 5: Figure S5), the mean amount of taxa significantly changing in abundance when using different DBs was dramatically higher in mice than in humans (61 vs. 16 % with MEGAN, 25 vs. 2 % with Unipept, respectively). Furthermore, the percentage of taxa consistently identified with MG-DBs in all subjects and never detected with UP-B, according to Unipept classification, was null in human and 15 % in mouse samples (0.5 and 3 % with MEGAN, respectively), with over 30 taxa uniquely detected with the contig-based DB. Hierarchical representation of differential taxa (graphically presented in cladograms of Additional file 6: Figure S6 and Additional file 7: Figure S7) revealed, again, few and reproducible distinctions in humans, counterbalanced by massive DB- and annotation tool-dependent biases in mice, involving (especially for MEGAN data) all main gut bacterial taxa, from phyla down to species. We finally took into account the *Firmicutes*/*Bacteroidetes* ratio, whose longitudinal change has been established as a synthetic measure of eubiosis/dysbiosis state within the gut microbiota [23, 24]. As illustrated in Fig. 5, we found a DB-related bias significantly influencing this parameter not only in mouse but also in human results, especially—and dramatically—when comparing UP-B to MG-DBs (up to tenfold higher ratio, when applying MEGAN to mouse data).

### Functional annotation of the mouse gut metaproteome is database-dependent

**Fig. 6:**
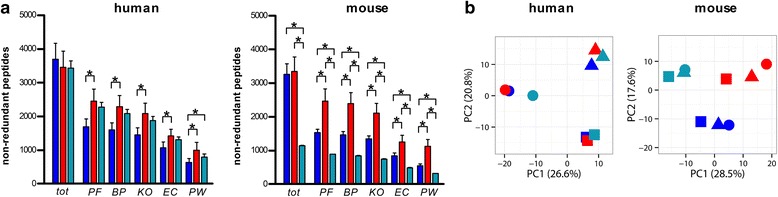
Functional annotation of human and mouse gut metaproteomic data obtained using different databases. DBs were made up of reads (*dark blue*, R-A) or contigs (*red*, C-A) from gut metagenomes of all subjects analyzed for each host species, or all bacterial sequences deposited in UniProt (*turquoise*, UP-B). **a** Histograms showing the mean number (*N* = 3, with *error bar* indicating standard error of the mean) of non-redundant peptides identified (*tot*) and annotated at different levels (*PF* protein family; *KO* KEGG ortholog; *EC* enzyme code; *BP* Gene Ontology Biological Process; *PW* pathway; *p < 0.05; **p < 0.01; ***p < 0.001; paired *t* test) in human (*left*) and mouse (*right*) samples upon a blastp against bacterial Swiss-Prot entries. **b** PCA plots of function abundances, with each *dot* indicating a different human (*left*) or mouse (*right*) subject

Finally, we focused on the functional annotation of metaproteomic data. In a preliminary test, we obtained a generally better functional annotation performance (in terms of absolute and relative amount of entries with complete annotation, at several levels) when blasting sequences against bacterial entries from Swiss-Prot, rather than blasting against the whole UniProt (i.e., Swiss-Prot+TrEMBL) DB, or retrieving KEGG or SEED functional information from MEGAN (data not shown). Functional information was therefore retrieved from Swiss-Prot. As shown in Fig. 6a, the contig-based DB significantly outperformed the read-based DB in terms of peptide annotation yield, while UP-B behaved slightly worse than the contig-based DB in human, and dramatically worse than both MG-DBs in mouse. Consistently with taxonomic data, human function abundances clustered according to individuals, whereas mouse data clustered according to the DB used (PCA plots in Fig. 6b). Furthermore, LEfSe differential analysis revealed that up to 15 and 39 % of the identified functions varied significantly when using different DB types in human and mouse, respectively (Additional file 8: Figure S8 and Additional file 9: Dataset S1). Interestingly, 60 protein families detected in all mice using the contig-based DB (16 % of total identification achieved using that DB) were not detected at all when using one or both the other DBs.

## Discussion

Many different data analysis strategies have been used in the gut metaproteomic studies published so far [16, 25–28], but a consensus has not been reached on which could be considered as the best performing DB search and annotation pipeline. To tackle this issue, here, we performed a systematic investigation aimed at evaluating the impact of sequence DB construction and annotation methods on gut metaproteomic results.

Since most gut microbiota investigations involve either human subjects or animal experimental models, we decided to analyze both human and mouse fecal samples. In view of the large heterogeneity observed in human populations [29] and a lower inter-individual diversity expected in syngenic mice [30], we hypothesized that human and mouse model metaproteomic studies would require dedicated data analysis strategies. In this respect, we demonstrate that the choice of performing shotgun metagenome sequencing to generate sample-matched (or, as suggested by these results, “population”-matched) DBs can considerably improve metaproteomic results, but this occurs with a considerably different magnitude in the two microbiota under study. In fact, a dramatically higher increase in terms of general identification yield and annotation depth can be observed in mouse when using a metagenomic DB instead of a UniProt-based DB. A possible explanation of this might reside in the considerably lower number of sequenced microbial strains among those colonizing the gut of mouse models, compared to the extensively studied microbes inhabiting the human gut. Consequently, the impact of this issue is expected to decrease as the number of sequences from the mouse gut metagenome grows in the years to come. When considering these results, special attention should be paid when selecting a DB for the analysis of a non-human gut microbiota, since uncritically applying data analysis pipelines optimized for human samples may lead to unexpectedly poor results.

Concerning the influence of metagenomic sequence assembly on metaproteomic analysis, our data also provide evidences that the choice between read- or contig-based DB considerably affects identification and annotation yields. Noteworthy, contigs clearly outperformed reads both for taxonomic and functional annotation yields, at least when annotation was carried out using blast-based methods. In particular, the association of contigs with MEGAN reached the best results in terms of taxonomic annotation yield, although in a previous study performed using lab-assembled microbial mixtures higher amounts of false positives were achieved when employing MEGAN for LCA classification instead of Unipept [15]. Clearly, the generation of DBs based on longer reads and contigs, stemming from the continuous improvement of pyrosequencing and sequencing by synthesis technologies, will further ameliorate both taxonomic and functional annotation yields.

With reference to UniProt-derived DBs, our data demonstrate that the advantage of performing a taxonomy-based sequence selection in order to obtain smaller-size DBs (essentially with the aim of reducing size-related FDR estimation issues) is clearly dependent on the particular combination of search engine and FDR calculation tool used. In more detail, merging information from Fig. 1 and Table 1 reveals that DB size-related issues appear to be stronger with X!Tandem/qvality, and almost negligible with Sequest-HT/Percolator. In many cases, however, the generation of a pseudo-metagenome based on taxonomic results from 16S rDNA analysis might be the preferred way—in the absence of matched shotgun metagenomes—to avoid huge analysis times (especially with MaxQuant) related to the use of the whole UniProt-bacteria DB. Besides size-related issues, taxonomic annotation was revealed to be strongly divergent when using a UniProt-based DB compared to MG-DBs (as exemplified by the *Firmicutes*/*Bacteroidetes* ratio), possibly due to biases in the number of deposited sequences within and among different phyla (as an example, *Firmicutes* entries are 3.5 times more represented than *Bacteroidetes* in the 2016_02 release of UniProt).

One of the main messages that can be inferred from our findings is that searching against different DB types and merging the related results can significantly deepen taxonomic and functional characterization of the gut metaproteome. The complementary nature of results, as well as the presence of DB-specific biases, suggests that the use of multiple DBs can likely lead to a more detailed and balanced picture of the biological activities exerted by intestinal microbial communities. Conversely, merging different types of sequences (e.g., reads and contigs) in a single DB led to lower identification yields compared to multiple parallel searches (data not shown). Although the advantage of using multiple search engines has been repeatedly demonstrated and highlighted [11, 31], the parallel use of multiple DBs has been much less frequently proposed as a systematic strategy [32, 33]. Similar considerations may be made with regard to taxonomic annotation tools, as a low amount of peptides were annotated both by MEGAN and Unipept (down to 15 % in mouse, data not shown); thus, most peptides were annotated by a single tool only, probably as a consequence of substantial differences between the two taxonomic classification algorithms. Under a general and practical perspective, the data analysis design of a gut microbiota investigation should aim for a compromise between sensitivity and information depth, on the one hand, and computational and time effort, on the other hand.

## Conclusions

This study contributes to identify host- and database-specific biases which need to be taken into account in a metaproteomic experiment, providing meaningful insights on how to design gut microbiota studies and to perform bioinformatic analysis of metaproteomic data. Specifically, the following recommendations can be made based on the data presented in this work: (i) coupling experimental metagenomic sequencing to metaproteomic analysis for DB construction purposes is mandatory when dealing with a non-human gut microbiota, but useful also for human studies; (ii) assembling reads into longer contigs may considerably enhance taxonomic and, even more, functional annotation; (iii) the use of multiple DBs and annotation tools has to be encouraged, even though this requires appropriate bioinformatic resources; (iv) the use of a “merged” DB, containing all metagenomic sequences from the population under study, is preferable over the use of sample-matched DBs; and (v) comparing results obtained with different DBs or tools should be carefully avoided, as it may lead to erroneous and unreliable conclusions. In keeping with these issues, the gut microbiota research community would greatly benefit from the development of specific bioinformatic applications facilitating browsing, selection, processing, merging, and annotation of sequencing data for metaproteomics.

## Methods

### Samples

Human stool samples were collected from four healthy Sardinian volunteers, who gave their informed consent for using the biological material for research purposes. Mouse stool samples (kindly provided by Dr. Michael Silverman, Mathis-Benoist Laboratory, Department of Microbiology and Immunobiology, Harvard Medical School, USA) were collected from three 10-week-old NOD mice. All samples were immediately stored at −80 °C until use. Then, samples were thawed at 4 °C, and from each of them two equal stool fragments (weighing approximately 250 and 30 mg for human and mouse samples, respectively) were collected: the first underwent DNA extraction for metagenomic analysis, and the second was subjected to protein extraction for metaproteomic analysis.

### DNA sample preparation and metagenome sequencing

DNA extraction was carried out using QIAamp Fast Stool Kit protocol (QIAGEN, Hilden, Germany). The extracted DNA was quantified on a Qubit 2.0 Fluorometer (Life Technologies, Grand Island, NY, USA), using the Qubit dsDNA High Sensitivity Assay Kit (Life Technologies). 16S rRNA gene amplification was performed using the universal primers 27F-1492R [34]. Two separate 16S rRNA gene amplification reactions were performed, pooled together, cleaned up using AMPure XP (Beckman Coulter, Brea, CA, USA) magnetic beads, and quantified with Qubit HS assay (Life Technologies). Libraries were constructed according the Illumina Nextera XT sample preparation protocol (Illumina, San Diego, CA, USA). Normalized sample libraries were pooled and subjected to the cluster generation step using the cBOT cluster generation station, according to the Illumina TruSeq Paired-End Cluster Kit protocol (Illumina). DNA sequencing was performed with the Illumina HiScanSQ sequencer, using the paired-end method and 93 cycles of sequencing. After sequencing, all reads were subjected to a demultiplexing step using Casava, v.1.8.2.

### Metagenome bioinformatics

16S data were processed using QIIME software package, v.1.8 [35]. The Illumina paired-reads were trimmed for the first 20 bp, and the sequences contaminated with Nextera adapters were identified using the UniVec database and removed. Therefore, the paired-reads with a minimum overlap of eight bases were merged and then filtered (first position quality score >15). Singletons were filtered with the same settings and added to the merged sequences that were clustered into OTUs at 97 % identity level against the Greengenes database (v.13_8) obtaining the BIOM table used to generate the pseudo-metagenomes. Concerning whole metagenome processing, raw reads were either filtered and clustered without assembly, or assembled de novo into contigs. In the first case, read processing was carried out using tools from the USEARCH suite v.8.0.1623 [36, 37]. Specifically, the following steps were performed sequentially: merging of paired reads (fastq_mergepairs command, setting parameters as follows: fastq_truncqual 3, fastq_minovlen 8, fastq_maxdiffs 0), quality filtering (fastq_filter command, with fastq_truncqual 15 and fastq_minlen 100), and sequence clustering (cluster_smallmem command, with 1 as identity threshold). In the second case, read assembly into contigs was carried out using Velvet v.1.2.10 [38], by setting 61 as k-mer length, 200 as insert length, and 300 as minimum contig length. ORF finding was carried out using FragGeneScan v.1.19 [39], training for Illumina sequencing reads with about 0.5 % error rate. Six-frame translation (6FT) was performed using the six-frame translation tool embedded in Max Quant v.1.5.2.8 [40], with 20 as minimum amino acid sequence length.

### Protein sample preparation and mass spectrometry analysis

Samples were resuspended by vortexing in SDS-based extraction buffer and then heated and subjected to a combination of bead-beating and freeze-thawing steps as detailed elsewhere [41]. Protein extracts were subjected to on-filter reduction, alkylation, and trypsin digestion according to the filter-aided sample preparation (FASP) protocol [42], with slight modifications detailed elsewhere [20, 43]. LC-MS/MS analysis was carried out using an LTQ-Orbitrap Velos mass spectrometer (Thermo Scientific, San Jose, CA, USA) interfaced with an UltiMate 3000 RSLCnano LC system (Thermo Scientific). The single-run 1D LC peptide separation was performed as previously described [41], loading 4 μg of peptide mixture per each sample and applying a 485-min (murine samples) or 153-min (human samples) separation gradient. The mass spectrometer was set up in a data dependent MS/MS mode, with higher-energy collision dissociation as the fragmentation method, as illustrated elsewhere [43].

### Metaproteome bioinformatics

Peptide identification was carried out using three bioinformatic platforms: MetaProteomeAnalyzer (MPA; v.1.0.6) [44], MaxQuant (MQ; v.1.5.2.8) [40], and Proteome Discoverer™ (PD; v.1.4.1.14; Thermo Scientific). For MPA analysis, X!Tandem [45] was used as search engine (precursor ion tolerance 10 ppm, fragment ion tolerance 0.02 Da, max missed cleavages 2, plus default search parameters) and qvality [46] as validation tool. For MQ analysis, carbamidomethylation of cysteine was set as fixed modification and oxidation of methionine as variable modification, and the other parameters were set as default. For PD analysis, Sequest-HT was used as search engine and Percolator [47] as validation tool, with all parameters set as described previously [20]. Two different false discovery rate thresholds were set for comparison, namely at 5 and 1 %.

DB construction was carried out as illustrated in Fig. 1. Human and murine MG-DBs were built on experimental sequences, after processing described in the “Metagenome bioinformatics” section. UniProt-based DBs were built on bacterial sequences retrieved from UniProt (Swiss-Prot+TrEMBL, release 2014_12). 16S-based DBs contained UniProt sequences (directly retrieved from the UniProt website) belonging to 17 families (namely, *Bacteroidaceae*, *Ruminococcaceae*, *Lachnospiraceae*, *Porphyromonadaceae*, *Paraprevotellaceae*, *Barnesiellaceae*, *Veillonellaceae*, *S24-7*, *Clostridiaceae*, *Rikenellaceae*, *Odoribacteraceae*, *Alcaligenaceae*, *Prevotellaceae*, *Christensenellaceae*, *Victivallaceae*, *Verrucomicrobiaceae*, *Erysipelotrichaceae*), 21 genera (namely, *Bacteroides*, *Ruminococcus*, *Faecalibacterium*, *Parabacteroides*, *Oscillospira*, *Lachnospira*, *Phascolarctobacterium*, *Coprococcus*, *Prevotella*, *Blautia*, *Sutterella*, *Clostridium*, *Dialister*, *Lachnobacterium*, *Ruminococcus*, *Butyricimonas*, *Odoribacter*, *Akkermansia*, *5-7N15*, *Anaerostipes*, *Roseburia*), or 19 species (namely, *Faecalibacterium prausnitzii*, *Bacteroides fragilis*, *Bacteroides plebeius*, *Parabacteroides distasonis*, *Bacteroides uniformis*, *Bacteroides ovatus*, *Akkermansia muciniphila*, *Bacteroides eggerthii*, *Bacteroides caccae*, *Bacteroides coprophilus*, *Ruminococcus gnavus*, *Bacteroides barnesiae*, *Ruminococcus bromii*, *Ruminococcus flavefaciens*, *Desulfovibrio D168*, *Ruminococcus callidus*, *Blautia producta*, *Prevotella melaninogenica*, *Prevotella nigrescens*) identified upon QIIME analysis of 16S rRNA sequencing data (abundance threshold 0.1 %). Proteomic iterative (PI) DBs were generated according to a taxonomy-based iterative strategy described earlier [15, 20]. Specifically, peptide sequences identified upon database search against UniProt-Bacteria were uploaded into the “Metaproteomics Analysis” module of the Unipept web application (v.2.5) [48] to carry out a taxonomic assignment based on the lowest common ancestor (LCA) approach, applying the following settings: “Equate I and L,” “Filter duplicate peptides,” and “Advanced missed cleavage handling”. Based on Unipept classification (abundance threshold 0.5 %), sequences belonging to specific families (namely, *Ruminococcaceae*, *Bacteroidaceae*, *Clostridiaceae*, *Eubacteriaceae*, *Lachnospiraceae*, *Prevotellaceae*, *Bifidobacteriaceae*, *Sutterellaceae*, *Acidaminococcaceae*, *Porphyromonadaceae*, *Akkermansiaceae*, and *Desulfovibrionaceae*, plus *Oscillospiraceae*, *Rikenellaceae*, and *Enterobacteriaceae* for PD analysis only), genera (namely, *Faecalibacterium*, *Bacteroides*, *Clostridium*, *Eubacterium*, *Subdoligranulum*, *Prevotella*, *Ruminococcus*, *Roseburia*, *Phascolarctobacterium*, *Bifidobacterium*, *Sutterella*, and *Akkermansia*, plus *Oscillibacter* and *Alistipes* for PD analysis only), or species (namely, *Faecalibacterium prausnitzii*, *Subdoligranulum variabile*, *Bacteroides plebeius*, *Bacteroides uniformis*, *Bacteroides vulgatus*, *Sutterella wadsworthensis*, *Bacteroides dorei*, *[Eubacterium] eligens*, *Akkermansia muciniphila*, *Bacteroides massiliensis*, *Ruminococcus bromii*, *Ruminococcus bicirculans*, *Bacteroides cellulosilyticus*, *Bilophila wadsworthia*, *Alistipes putredinis*, and *Phascolarctobacterium succinatutens*, plus *Parasutterella excrementihominis* for MPA analysis only, and *Bacteroides thetaiotaomicron* for MQ and PD analyses only) were retrieved from UniProt.

Taxonomic annotation of metaproteomic data was carried out using MEGAN (v.5.9) [49] and Unipept. For MEGAN analysis, read, contig, and protein sequences (identified from each respective DB) were subjected to blastp search against the NCBI-nr DB (e-value threshold 10^−5^), and LCA was performed on blast results using default parameters. For Unipept analysis, peptide sequences were uploaded in the Metaproteomics Analysis module and classified using the above described settings.

Functional annotation was accomplished by blastp search (e-value threshold 10^−5^) against bacterial sequences from the UniProt/Swiss-Prot database (release 2014_12) and subsequent retrieval of protein family, KEGG orthologous groups, enzyme codes, Gene Ontology biological processes, and pathway information associated with each UniProt/Swiss-Prot accession number [50].

Shannon’s index for alpha diversity estimation was calculated according to established methods [51]. Differentially abundant features were identified by linear discriminant analysis and effect-size calculation using LEfSe [20, 52], with the following significance thresholds: log LDA score >2; alpha-value <0.05. Cladograms were generated by LEfSe and modified using Inkscape (https://inkscape.org), and histograms and dot plots were generated using GraphPad Prism (v.5.03), and Venn diagrams were generated using Venny (v.2.1.0; http://bioinfogp.cnb.csic.es/tools/venny/index.html) and Venn Diagram Plotter (v.1.5.5228; https://omics.pnl.gov/software/venn-diagram-plotter). Principal component analysis (with singular value decomposition and imputation of missing data) was carried out using ClustVis [53]. Statistical significance of differences was assessed by applying a paired *t* test.

## Additional files

Additional file 1: Figure S1. (A) The influence of the ORF finding approach on metagenomic databases. Venn diagrams indicate overlap among non-redundant peptide identifications obtained using ORFs found by FragGeneScan (FGS) or six-frame translation (6FT) as sequence databases. Percentage increase related to the use of a 6FT database in addition to the corresponding FGS database is shown on the bottom-right of each Venn diagram. Metagenomic databases derive from a 6-Mbps metagenome. Peptide identifications were obtained at 5 % FDR, using three different bioinformatic platforms (left, MetaProteomeAnalyzer; middle, MaxQuant; right, Proteome Discoverer) with the corresponding (and above indicated) search engines and peptide validation tools. (B) Comparison between reads- and contigs-based metagenomic databases at different sequencing depths. Venn diagrams indicate overlap among non-redundant peptide identifications obtained using reads (R) or assembled contigs (C) as sequence databases. ORFs from both reads and contigs were found by FragGeneScan (F). Metagenome sequencing depth was 18 (top), 6 (middle), or 3 (bottom) Mbps. Percentage increase related to the use of contigs-based database in addition to the corresponding reads-based database is shown in red on the bottom-right of each Venn diagram, while percentage increase related to the use of reads-based database in addition to the corresponding contigs-based database is shown in blue on the bottom-left of each Venn diagram. Peptide identifications were obtained at 5 % FDR, using three different bioinformatic platforms (left, MetaProteomeAnalyzer; middle, MaxQuant; right, Proteome Discoverer) with the corresponding (and above indicated) search engines and peptide validation tools. (TIF 2050 KB) Additional file 2: Figure S2. The influence of taxonomic filter level on the 16S-based (16S, top) and proteomic iterative (PI, bottom) databases. Venn diagrams indicate overlap among non-redundant peptide identifications obtained using UniProt sequences belonging to all families (F), genera (G), or species (S) detected after 16S rDNA gene sequencing and QIIME taxonomic assignment (16S; taxon abundance threshold >0.1 %), or a preliminary search against UniProt Bacteria and peptide taxonomic assignment using Unipept (PI; taxon abundance threshold >0.5 %). Peptide identifications were obtained at 5 % FDR, using three different bioinformatic platforms (left, MetaProteomeAnalyzer; middle, MaxQuant; right, Proteome Discoverer) with the corresponding (and above indicated) search engines and peptide validation tools. (TIF 1580 KB) Additional file 3: Figure S3. Complementarity between reads-based database and other database types. Venn diagrams indicate overlap among non-redundant peptide identifications obtained using different sequence databases (see Additional file 1: Figure S1 and Additional file 2: Figure S2 for further details). Percentage increase related to the use of a given database in addition to the counterpart is shown on the bottom, on the same side and in the same color. Peptide identifications were obtained at 5 % FDR, using three different bioinformatic platforms (left, MetaProteomeAnalyzer; middle, MaxQuant; right, Proteome Discoverer) with the corresponding (and above indicated) search engines and peptide validation tools. (TIF 1780 KB) Additional file 4: Figure S4. Alpha-diversity values measured in human (A) and mouse (B) samples according to Shannon’s index. Dark blue, database comprising reads from gut metagenomes of all subjects analyzed for that host species; red, database comprising contigs assembled from gut metagenomes of all subjects analyzed for that host species; turquoise, all bacterial sequences deposited in UniProt (UP-B). **p* < 0.05; ***p* < 0.01. (TIF 349 KB) Additional file 5: Figure S5. Histograms illustrating the number of taxa identified with the three DBs in human (left) and mouse (right) samples, distinguishing qualitatively differential (black), quantitatively differential (white) and non-differential (grey) taxa. (TIF 648 KB) Additional file 6: Figure S6. Cladograms illustrating human gut microbiota taxa detected with differential abundance when using different sequence databases. Spectral counting data obtained upon Sequest-HT/Percolator search (1 % FDR) and MEGAN (left) or Unipept (right) lowest common ancestor (LCA) taxonomic classification were uploaded into LEfSe for differential analysis and cladogram construction. Taxa with higher abundance related to a given database are marked with the specific database color (dark blue, reads-based MG-DB; red, contigs-based MG-DB; turquoise, UniProt Bacteria). (TIF 3450 KB) Additional file 7: Figure S7. Cladograms illustrating mouse gut microbiota taxa detected with differential abundance when using different sequence databases. Spectral counting data obtained upon Sequest-HT/Percolator search (1 % FDR) and MEGAN (left) or Unipept (right) lowest common ancestor (LCA) taxonomic classification were uploaded into LEfSe for differential analysis and cladogram construction. Taxa with higher abundance related to a given database are marked with the specific database color (dark blue, reads-based MG-DB; red, contigs-based MG-DB; turquoise, UniProt Bacteria). (TIF 7330 KB) Additional file 8: Figure S8. Histograms illustrating the number of protein families identified with the three DBs in human (left) and mouse (right) samples, distinguishing qualitatively differential (black), quantitatively differential (white) and non-differential (gray) features. (TIF 247 KB) Additional file 9: Dataset S1. Lists of protein families detected with significantly varying abundance when using different DB types in human and mouse samples. (XLSX 28.0 KB)

## Abbreviations

DB: Database
FASP: Filter-aided sample preparation
LCA: Lowest common ancestor
MG: Metagenomic
MPA: MetaProteomeAnalyzer
MQ: MaxQuant
MS: Mass spectrometry
PCA: Principal component analysis
PD: Proteome Discoverer
PI: Proteomic iterative
UP-B: UniProt Bacteria
