# Supplementary figures and images for: The impact of sequence database choice on metaproteomic results in gut microbiota studies
Source: Microbiome. 2016 Sep 27;4:51. doi: 10.1186/s40168-016-0196-8 (PMC5037606; doi:10.1186/s40168-016-0196-8)

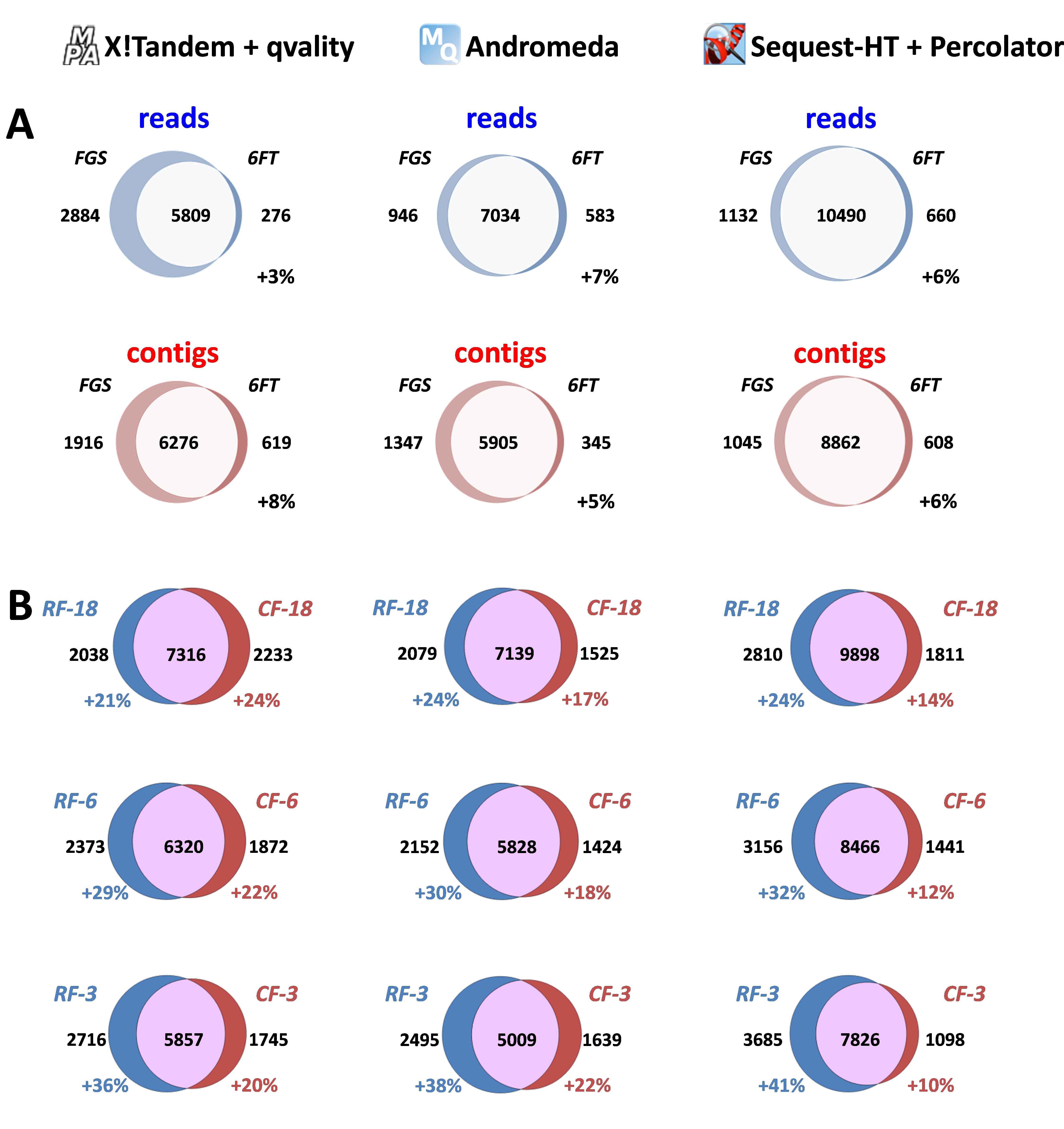

Supplement: Additional file 1: Figure S1. — (A) The influence of the ORF finding approach on metagenomic databases. Venn diagrams indicate overlap among non-redundant peptide identifications obtained using ORFs found by FragGeneScan (FGS) or six-frame translation (6FT) as sequence databases. Percentage increase related to the use of a 6FT database in addition to the corresponding FGS database is shown on the bottom-right of each Venn diagram. Metagenomic databases derive from a 6-Mbps metagenome. Peptide identifications were obtained at 5 % FDR, using three different bioinformatic platforms (left, MetaProteomeAnalyzer; middle, MaxQuant; right, Proteome Discoverer) with the corresponding (and above indicated) search engines and peptide validation tools. (B) Comparison between reads- and contigs-based metagenomic databases at different sequencing depths. Venn diagrams indicate overlap among non-redundant peptide identifications obtained using reads (R) or assembled contigs (C) as sequence databases. ORFs from both reads and contigs were found by FragGeneScan (F). Metagenome sequencing depth was 18 (top), 6 (middle), or 3 (bottom) Mbps. Percentage increase related to the use of contigs-based database in addition to the corresponding reads-based database is shown in red on the bottom-right of each Venn diagram, while percentage increase related to the use of reads-based database in addition to the corresponding contigs-based database is shown in blue on the bottom-left of each Venn diagram. Peptide identifications were obtained at 5 % FDR, using three different bioinformatic platforms (left, MetaProteomeAnalyzer; middle, MaxQuant; right, Proteome Discoverer) with the corresponding (and above indicated) search engines and peptide validation tools. (TIF 2050 KB) [file 40168_2016_196_MOESM1_ESM.tif]

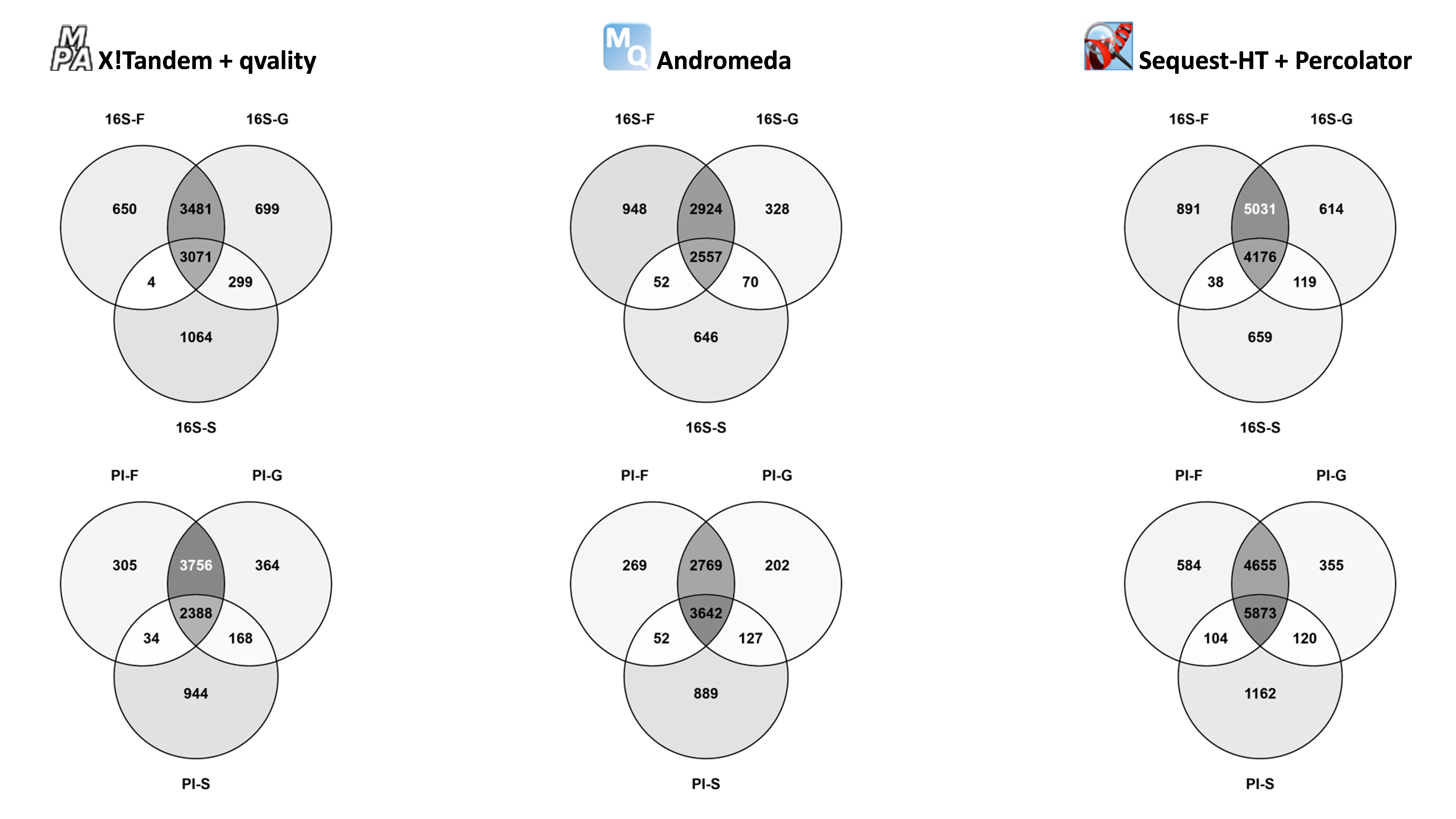

Supplement: Additional file 2: Figure S2. — The influence of taxonomic filter level on the 16S-based (16S, top) and proteomic iterative (PI, bottom) databases. Venn diagrams indicate overlap among non-redundant peptide identifications obtained using UniProt sequences belonging to all families (F), genera (G), or species (S) detected after 16S rDNA gene sequencing and QIIME taxonomic assignment (16S; taxon abundance threshold >0.1 %), or a preliminary search against UniProt Bacteria and peptide taxonomic assignment using Unipept (PI; taxon abundance threshold >0.5 %). Peptide identifications were obtained at 5 % FDR, using three different bioinformatic platforms (left, MetaProteomeAnalyzer; middle, MaxQuant; right, Proteome Discoverer) with the corresponding (and above indicated) search engines and peptide validation tools. (TIF 1580 KB) [file 40168_2016_196_MOESM2_ESM.tif]

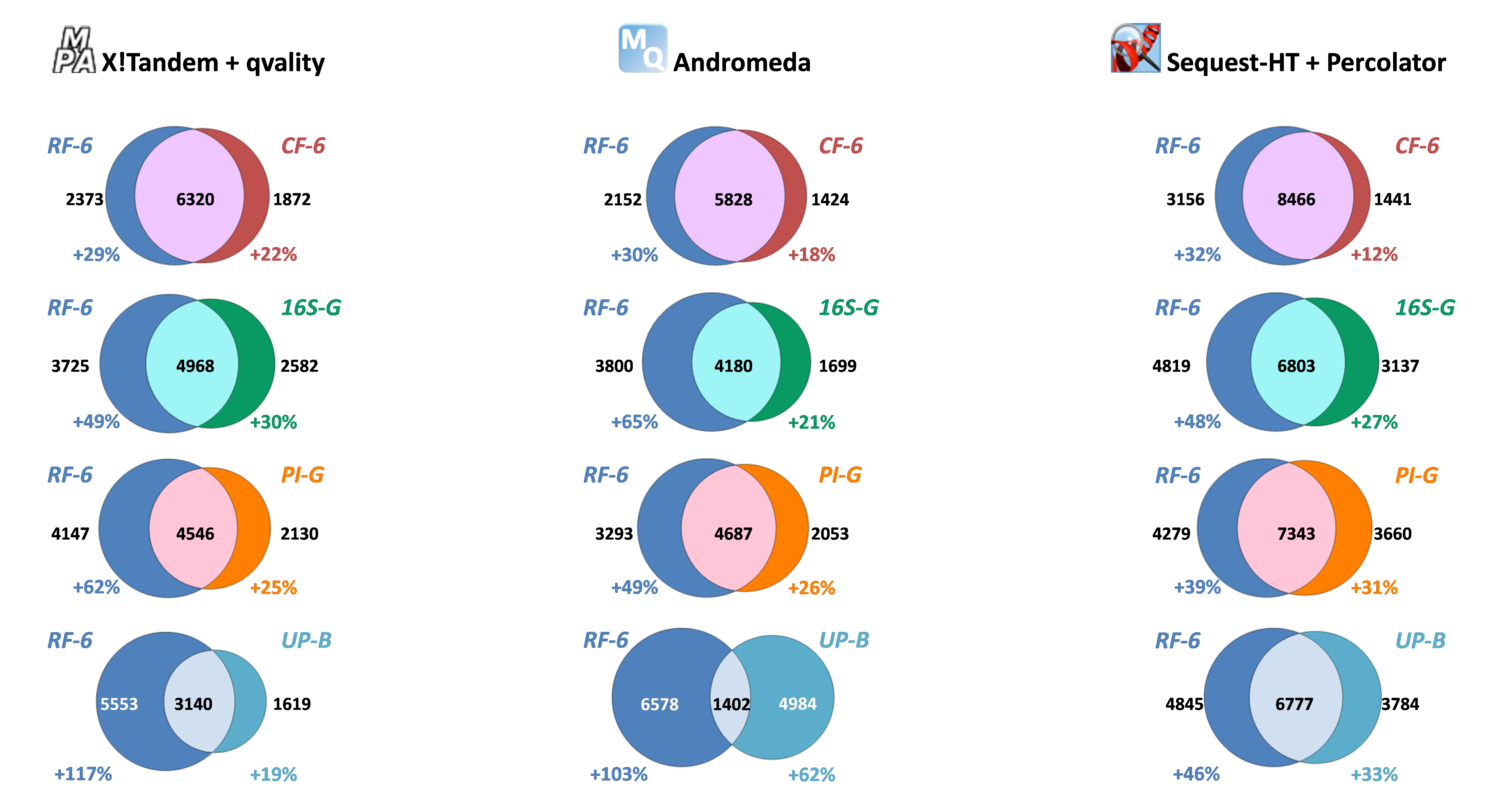

Supplement: Additional file 3: Figure S3. — Complementarity between reads-based database and other database types. Venn diagrams indicate overlap among non-redundant peptide identifications obtained using different sequence databases (see Additional file 1: Figure S1 and Additional file 2: Figure S2 for further details). Percentage increase related to the use of a given database in addition to the counterpart is shown on the bottom, on the same side and in the same color. Peptide identifications were obtained at 5 % FDR, using three different bioinformatic platforms (left, MetaProteomeAnalyzer; middle, MaxQuant; right, Proteome Discoverer) with the corresponding (and above indicated) search engines and peptide validation tools. (TIF 1780 KB) [file 40168_2016_196_MOESM3_ESM.tif]

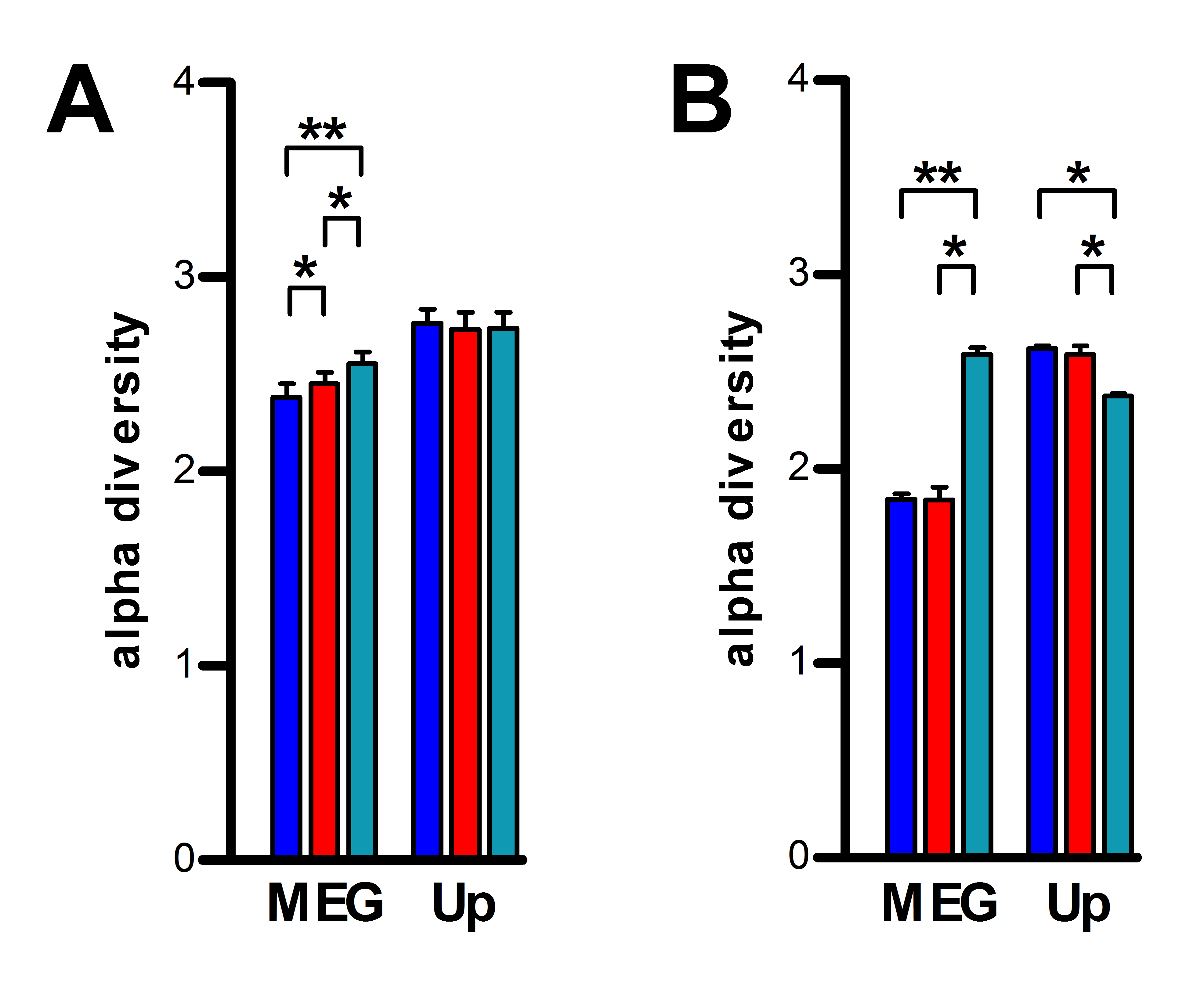

Supplement: Additional file 4: Figure S4. — Alpha-diversity values measured in human (A) and mouse (B) samples according to Shannon’s index. Dark blue, database comprising reads from gut metagenomes of all subjects analyzed for that host species; red, database comprising contigs assembled from gut metagenomes of all subjects analyzed for that host species; turquoise, all bacterial sequences deposited in UniProt (UP-B). *p < 0.05; **p < 0.01. (TIF 349 KB) [file 40168_2016_196_MOESM4_ESM.tif]

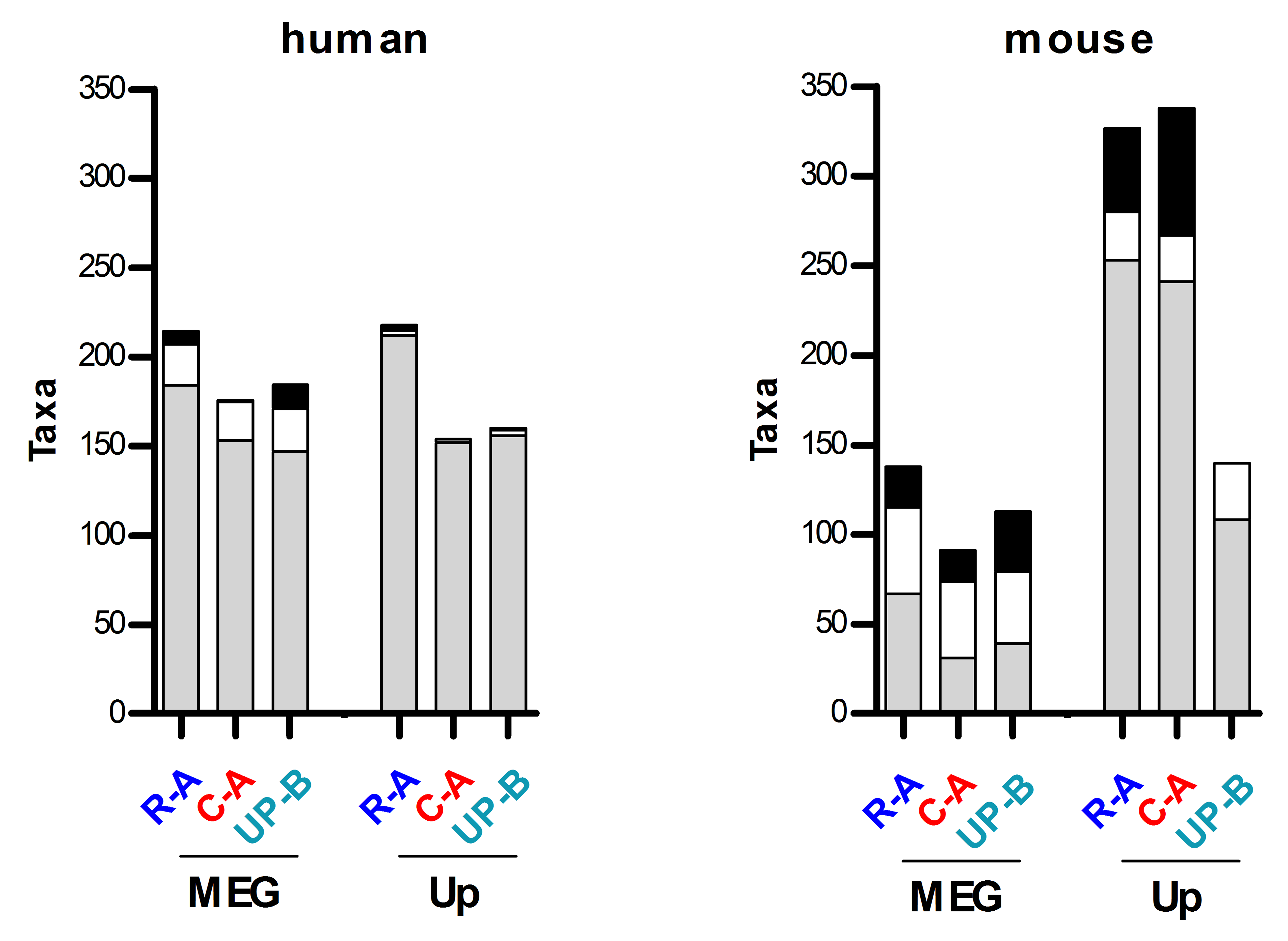

Supplement: Additional file 5: Figure S5. — Histograms illustrating the number of taxa identified with the three DBs in human (left) and mouse (right) samples, distinguishing qualitatively differential (black), quantitatively differential (white) and non-differential (grey) taxa. (TIF 648 KB) [file 40168_2016_196_MOESM5_ESM.tif]

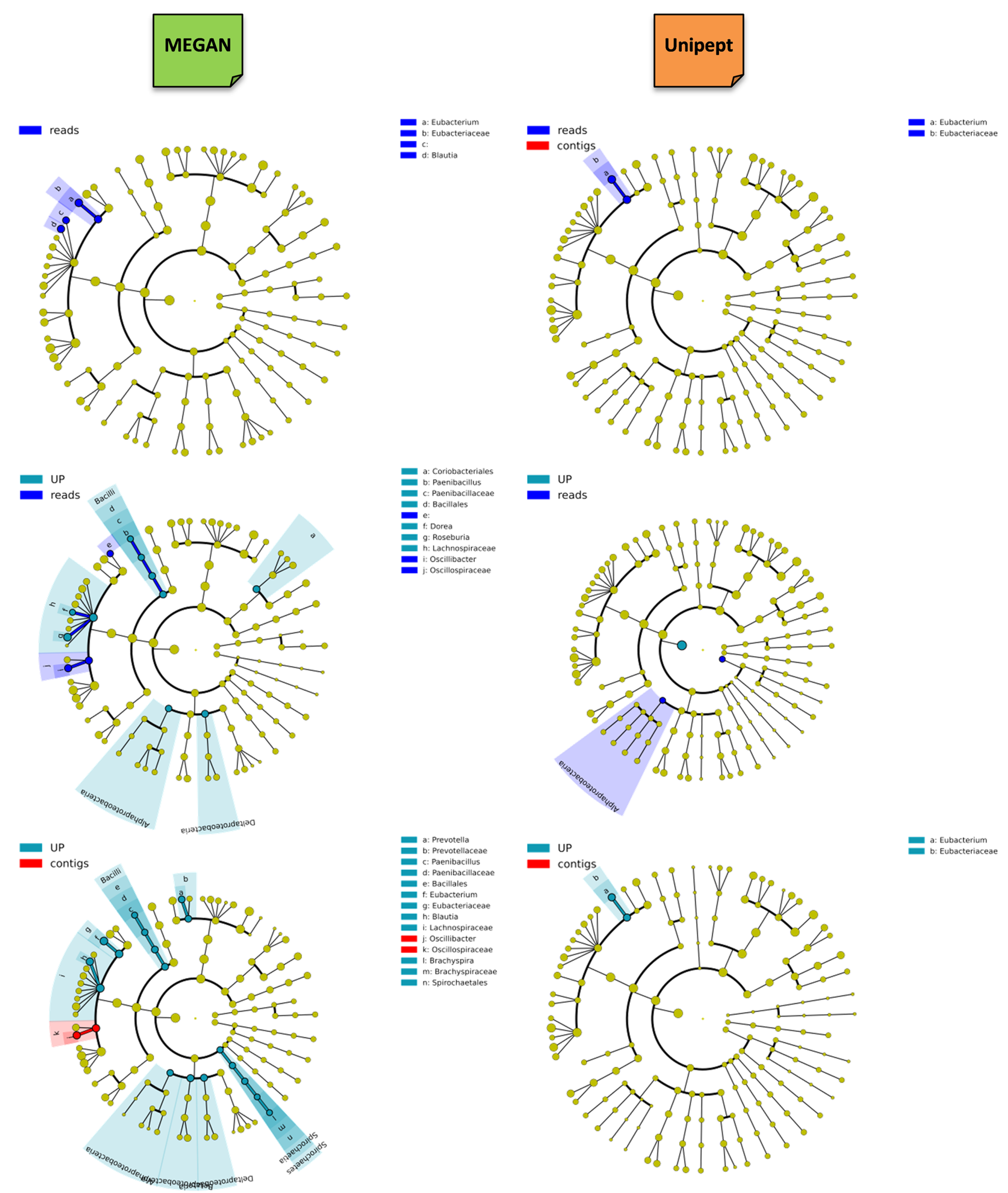

Supplement: Additional file 6: Figure S6. — Cladograms illustrating human gut microbiota taxa detected with differential abundance when using different sequence databases. Spectral counting data obtained upon Sequest-HT/Percolator search (1 % FDR) and MEGAN (left) or Unipept (right) lowest common ancestor (LCA) taxonomic classification were uploaded into LEfSe for differential analysis and cladogram construction. Taxa with higher abundance related to a given database are marked with the specific database color (dark blue, reads-based MG-DB; red, contigs-based MG-DB; turquoise, UniProt Bacteria). (TIF 3450 KB) [file 40168_2016_196_MOESM6_ESM.tif]

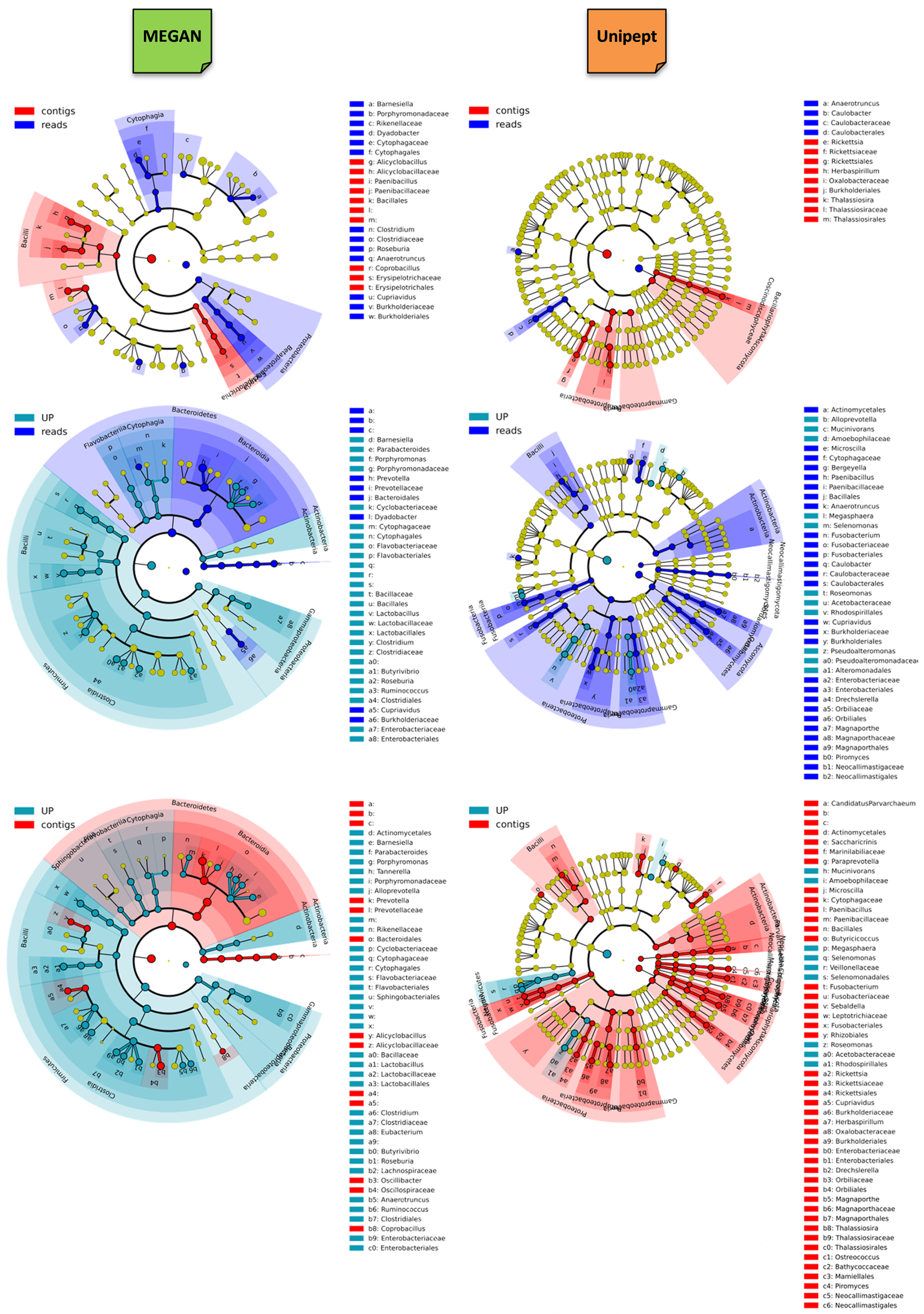

Supplement: Additional file 7: Figure S7. — Cladograms illustrating mouse gut microbiota taxa detected with differential abundance when using different sequence databases. Spectral counting data obtained upon Sequest-HT/Percolator search (1 % FDR) and MEGAN (left) or Unipept (right) lowest common ancestor (LCA) taxonomic classification were uploaded into LEfSe for differential analysis and cladogram construction. Taxa with higher abundance related to a given database are marked with the specific database color (dark blue, reads-based MG-DB; red, contigs-based MG-DB; turquoise, UniProt Bacteria). (TIF 7330 KB) [file 40168_2016_196_MOESM7_ESM.tif]

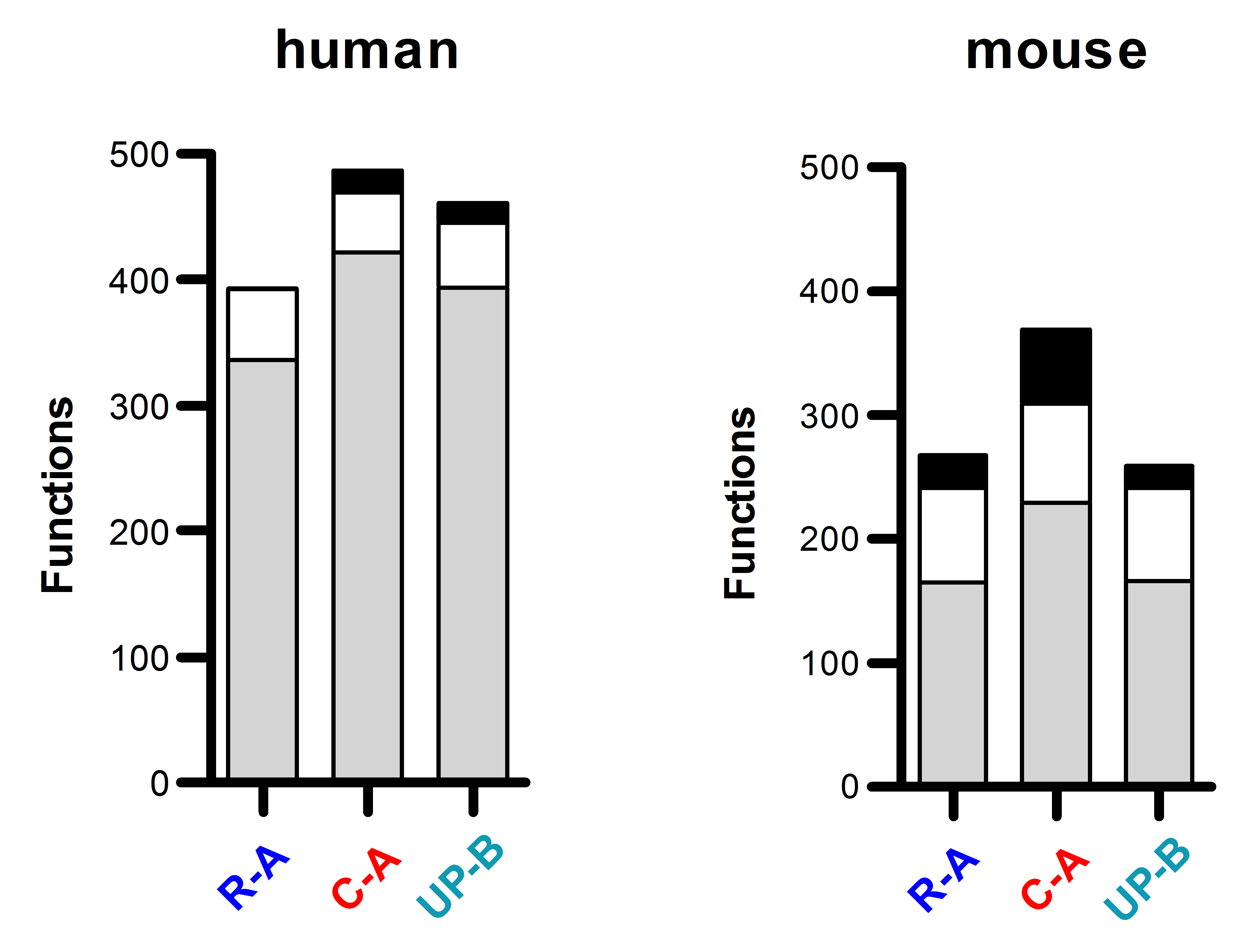

Supplement: Additional file 8: Figure S8. — Histograms illustrating the number of protein families identified with the three DBs in human (left) and mouse (right) samples, distinguishing qualitatively differential (black), quantitatively differential (white) and non-differential (gray) features. (TIF 247 KB) [file 40168_2016_196_MOESM8_ESM.tif]
